# Dyspnea and Exercise Limitation in Mild COPD: The Value of CPET

**DOI:** 10.3389/fmed.2020.00442

**Published:** 2020-08-13

**Authors:** Matthew D. James, Kathryn M. Milne, Devin B. Phillips, J. Alberto Neder, Denis E. O'Donnell

**Affiliations:** ^1^Respiratory Investigation Unit, Department of Medicine, Queen's University, Kingston, ON, Canada; ^2^Clinician Investigator Program, University of British Colombia, Vancouver, BC, Canada; ^3^Laboratory of Clinical and Exercise Physiology, Department of Medicine, Queen's University, Kingston, ON, Canada

**Keywords:** cardiopulmonary exercise testing, chronic obstructive pulmonary disease, dyspnea, neural drive, respiratory mechanics, gas exchange

## Abstract

The majority of smokers with chronic obstructive pulmonary disease (COPD) have mild airflow limitation as determined by simple spirometry. Although small airway dysfunction is the hallmark of COPD, many studies attest to complex heterogeneous physiological impairments beyond increased airway resistance. These impairments are related to inflammation of lung parenchyma and its microvasculature, which is obscured by simple spirometry. Recent studies using advanced radiological imaging have highlighted significant structural abnormalities in smokers with relatively preserved spirometry. These important studies have generated considerable interest and have reinforced the pressing need to better understand the physiological consequences of various morphological abnormalities, and their impact on the clinical outcomes and natural history of COPD. The overarching objective of this review is to provide a concise overview of the importance and utility of cardiopulmonary exercise testing (CPET) in clinical and research settings. CPET uniquely allows evaluation of integrated abnormalities of the respiratory, cardio-circulatory, metabolic, peripheral muscle and neurosensory systems during increases in physiologic stress. This brief review examines the results of recent studies in mild COPD that have uncovered consistent derangements in pulmonary gas exchange and development of “restrictive” dynamic mechanics that together contribute to exercise intolerance. We examine the evidence that compensatory increases in inspiratory neural drive from respiratory control centers are required during exercise in mild COPD to maintain ventilation commensurate with increasing metabolic demand. The ultimate clinical consequences of this high inspiratory neural drive are earlier onset of critical respiratory mechanical constraints and increased perceived respiratory discomfort at relatively low exercise intensities.

## Introduction

Chronic obstructive pulmonary disease (COPD) is a progressive and debilitating inflammatory disease of the airways, alveoli, and microvasculature. Patients classified in the mild COPD stage by Global Initiative for Chronic Obstructive Lung Disease (GOLD) criteria represent the majority of total patients with COPD, with an estimated global prevalence of 7–11% in adults over 40 years of age (1–3). Such patients with mild COPD have an increased risk of morbidity and mortality compared to healthy non-smokers, and have reduced health-related quality of life (4). The most commonly reported symptom in patients with mild COPD is dyspnea (breathlessness), defined by a 2012 American Thoracic Society (ATS) statement as “a subjective experience of breathing discomfort that consists of qualitatively distinct sensations that vary in intensity” (5). Dyspnea is particularly troublesome during exertion in patients with mild COPD, and is often disproportionate to the degree of airflow limitation (6). Further, dyspnea upon exertion has been linked to exercise intolerance in these patients (6, 7). The effective management of this complex and multifactorial symptom, exercise intolerance and the associated poor health status, remains a substantial challenge for caregivers. Interest in systematic evaluation of the heterogeneous physiological derangements of mild COPD has mounted since the discovery of widespread structural abnormalities, quantified by imaging, in large numbers of smokers who have normal spirometry as well as in those who meet GOLD diagnostic spirometric criteria (8, 9). In this context, our understanding of the nature of physiological impairment and its negative consequences (dyspnea and exercise intolerance) in mild COPD has been considerably enhanced by the use of non-invasive cardiopulmonary exercise testing (CPET) using treadmill or cycle ergometry, which allows for the measurement of subjective (i.e., dyspnea measured by Borg scale) and physiologic (breath by breath measures of ventilatory and metabolic) parameters during a standardized physical stimulus or stress (6, 7, 10–12). Conventional CPET is particularly valuable in mild COPD where apparently disproportionate dyspnea and reduced exercise tolerance remain unexplained after routine spirometry. This review will outline evolving concepts of the physiological underpinnings of dyspnea and exercise intolerance in mild COPD, and highlight the clinical utility of CPET.

For the purpose of this review, we propose that mild COPD refer to patients with the following criteria: (1) relevant long-term exposure to tobacco smoke (i.e., current or ex-smoker with significant smoking history), (2) persistent symptoms of dyspnea, cough, and/or sputum production that are not explained by other respiratory disorder, (3) a post-bronchodilator forced expiratory volume in 1 s (FEV_1_) to forced vital capacity (FVC) ratio <0.7, with an FEV_1_ >80% predicted, measured by simple spirometry. Essentially, we focused our review on physiological exercise studies undertaken in smokers meeting GOLD Stage 1B COPD criteria.

## Exertional Dyspnea in Copd: Current Constructs

**Figure 1:**
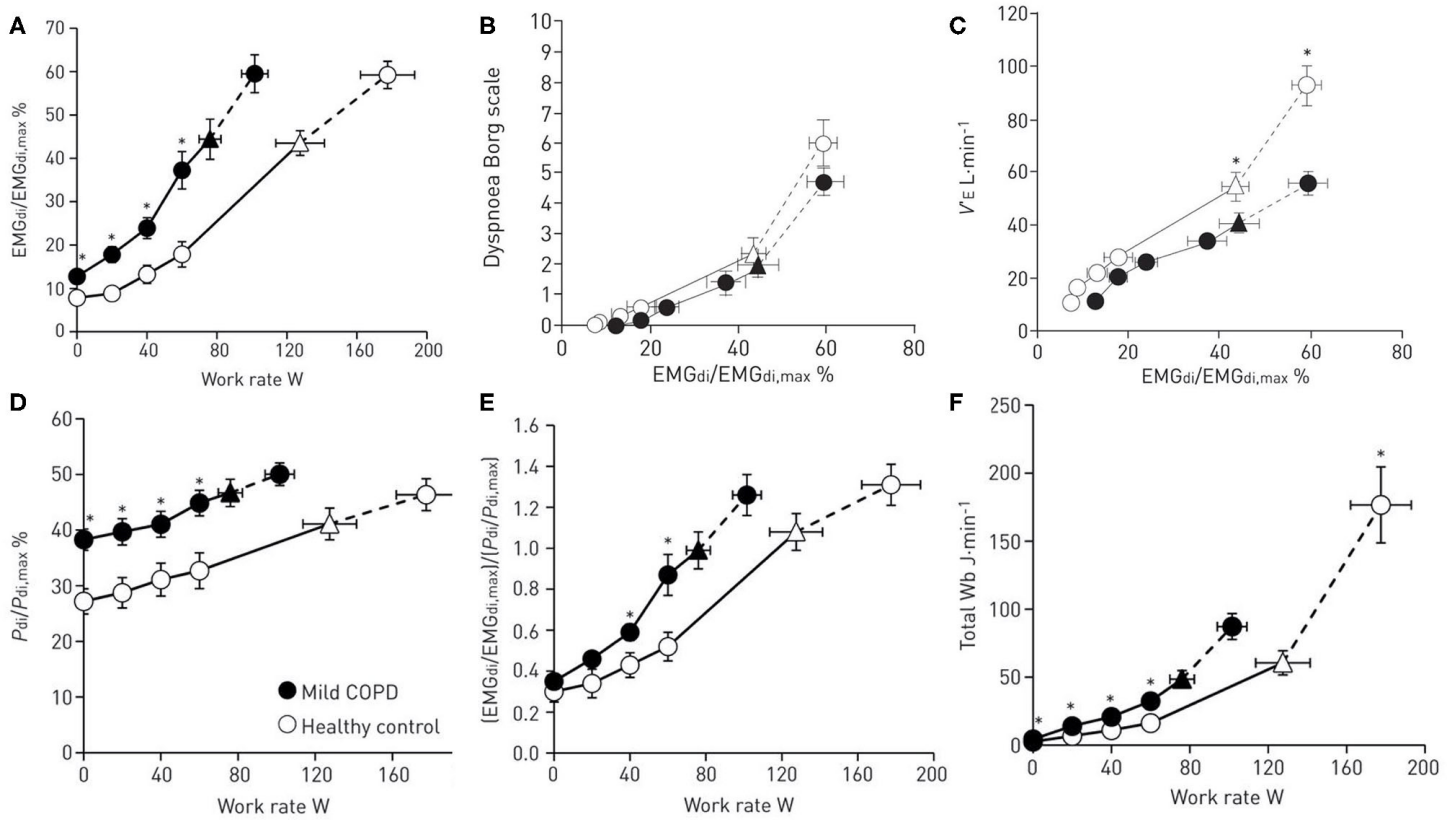
Ventilatory drive and respiratory mechanical response to exercise in patients with mild chronic obstructive pulmonary disease (COPD) compared to healthy controls. **(A)** The ratio of diaphragmatic electromyography (EMGdi) to maximal EMGdi (EMGdi,max) relative to work rate, **(B)** dyspnea intensity (measured by modified 10-point Borg scale) relative to the ratio of EMGdi/EMGdi,max **(C)** ventilation (V_E_) relative to the ratio of EMGdi/EMGdi,max **(D)** the ratio of transdiaphragmatic pressure (Pdi) relative to maximal Pdi (Pdi,max) relative to work rate **(E)** neuromechanical dissociation (represented as the ratio of EMGdi/EMGdi,max to Pdi/Pdi,max) relative to work rate **(F)** total work of breathing relative to work rate. Data are presented as Mean ± SEM. Triangles represent the tidal volume/minute ventilation inflection point. Reproduced with permission of the © ERS 2020: Guenette et al. (7).

Dyspnea is believed to arise from an imbalance between inspiratory neural drive (IND) to breathe and the capacity of the respiratory system to respond (13). This imbalance is variably termed demand-capacity imbalance, efferent-afferent dissociation, neuromechanical, or neuromuscular dissociation. This theory is supported by studies that demonstrate a strong association between the rise in dyspnea intensity during exercise and simultaneous increase in several physiologic ratios (IND, ventilation and muscular effort, all relative to their maximal values) which collectively reflect demand-capacity imbalance of the respiratory system [Figure 1; (15–17)]. Together, these studies support the notion that dyspnea increases during exercise as a function of increasing IND (from bulbo-pontine and cortical respiratory control centers) in the face of an ever-decreasing capacity of the respiratory system to appropriately respond, because of significant mechanical constraints.

## Mechanisms of Increased Inspiratory Neural Drive in Mild Copd

Recent studies in mild COPD have shown that dyspnea during exercise is associated with disproportionately increased IND (as measured by diaphragm electromyography, EMGdi) compared with healthy controls [Figure 1A; (7)]. The abnormally high IND in mild COPD is mainly attributed to: (1) reduced ventilatory efficiency during exercise (i.e., increased ventilation relative to carbon dioxide production, V_E_/VCO_2_), and/or (2) progressive expiratory flow limitation (EFL) and abnormal dynamic breathing mechanics (6, 10, 11).

### Pulmonary Gas Exchange Abnormalities

The increased IND during exercise in COPD patients is the result of variable perturbation of both chemical and respiratory mechanical factors (18, 19). In both health and COPD, IND increases as carbon dioxide output (VCO_2_) increases during exercise in response to greater energy expenditure and metabolic demand (20). Thus, exercise hyperpnea is closely linked to pulmonary CO_2_ gas exchange. Tobacco-related injury of the lung parenchyma, small airways, and microvasculature in mild COPD leads to heterogeneous ventilation-perfusion mismatch and significant abnormalities in pulmonary gas exchange (11, 21–23). In mild COPD during exercise, V_E_/VCO_2_, physiological dead space (V_D_), the dead space to tidal volume ratio (V_D_/V_T_), and alveolar ventilation (V_A_) are elevated when compared with health (11). When combined with EFL, these elevated ventilatory requirements lead to accelerated progression of dynamic mechanical constraints, dyspnea and reduced exercise tolerance (10, 11). Additionally, the associated tachypnea and shallow breathing pattern further increase the dead space to tidal volume ratio (V_D_/V_T_) (11).

Recently, attenuated pulmonary capillary perfusion has been demonstrated at rest and during exercise in mild COPD (11, 21, 24, 25). Indeed, several studies utilizing various radiological imaging techniques have confirmed important structural and functional abnormalities of the small airways and microvasculature in smokers with normal spirometry and in patients with mild COPD (8, 9, 23, 26). Quantitative high-resolution computed tomography (CT) imaging has shown that patients with mild COPD can have significant emphysema, pulmonary gas trapping, small airway thickening, and vascular abnormalities (8). Studies using contrast-enhanced MRI have provided evidence of significant pulmonary capillary perfusion abnormalities suggesting tobacco smoke-induced vasculopathy may be present even in smokers with mild spirometric abnormalities (9, 23).

**Figure 2:**
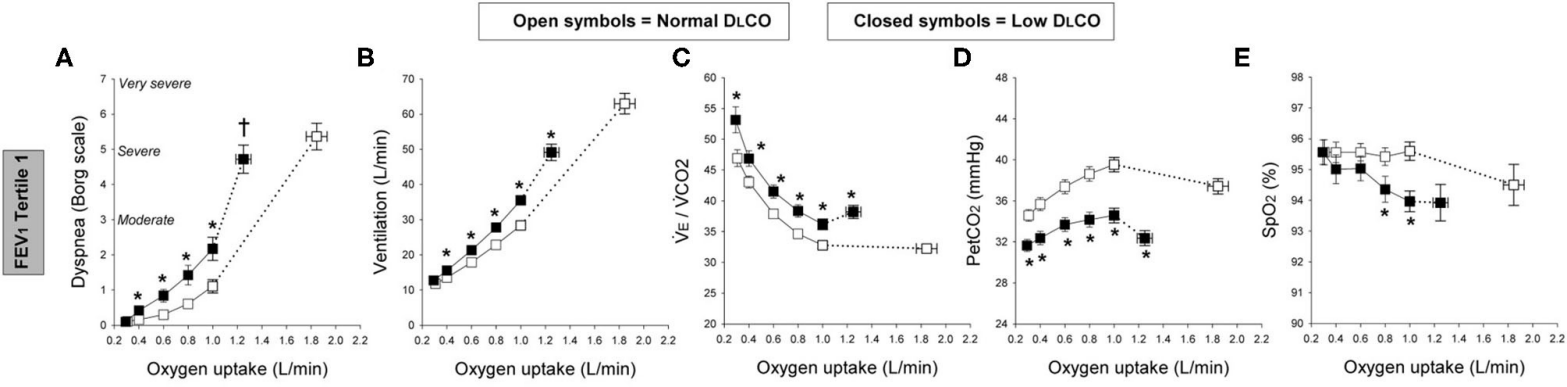
Exertional dyspnea intensity (Borg scale; **A**), ventilation **(B)**, ventilatory equivalent for carbon-dioxide (V_E_/VCO2; **C**), partial pressure of end-tidal CO2 (P_ET_CO_2_; **D**), and oxygen saturation by pulse oximetry (SpO_2_; **E**); all for a given oxygen uptake during symptom-limited incremental cycle exercise in chronic obstructive pulmonary disease patients with normal single-breath diffusing capacity of the lung for carbon monoxide (DLCO; open symbols) and those with low DLCO (closed symbols) in the first tertile of forced expiratory volume in 1 s (FEV1). Tertile 1 (FEV1 > 73.5%predicted, square symbols). Data are means ± SE. *P < 0.05, normal vs. low DLCO.^†^*P* < 0.05, difference in dyspnea/oxygen uptake slope between normal and low DLCO. Elbehairy et al. (12).

Moreover, new studies have shown that a low resting diffusing capacity for carbon monoxide (DLCO)—which provides a window into the microvasculature—is associated with ventilatory inefficiency (high V_E_/VCO_2_) and increased exertional dyspnea in smokers with and without airway obstruction (11, 24, 27). It has become clear that DLCO reliably evaluates the integrity of the alveolar-capillary interface in mild COPD where significant maldistribution of alveolar ventilation is absent, unlike more advanced disease (28). Elbehairy et al. demonstrated consistently higher ratings of dyspnea intensity and reduced exercise tolerance in COPD patients with a DLCO below the lower limit of normal (< LLN), when compared with patients with preserved DLCO [Figure 2A; (12)]. The higher dyspnea ratings and earlier exercise termination in the low DLCO groups were linked to significantly greater ventilatory inefficiency (i.e., high V_E_/VCO_2_) mainly reflecting higher physiological dead space [Figure 2C; (11, 12)]. Accordingly, compromised CO_2_ elimination due to ventilation-perfusion inequalities and resultant increased chemo-stimulation gave rise to high ventilatory requirements (Figure 2B) that accelerates dynamic mechanical constraints (breathing pattern abnormalities and critical tidal volume constraints) at lower exercise intensities than patients with preserved DLCO (12).

Other chemical factors which are believed to contribute to increased IND during exercise in more advanced COPD are less likely to be important in mild COPD. These include: (1) increased chemosensitivity and a lower regulated level of arterial pCO_2_ (21, 29–32); (2) increased chemoreceptor stimulation [due to critical arterial hypoxemia, low mixed venous O_2_ returning to areas in the lung with low ventilation-perfusion (V/Q) ratios] (11, 33–36); (3) skeletal muscle deconditioning (manifesting as metabolic acidosis at relatively low VO_2_) which result in increased afferent ergoreceptor activation (due to reduced O_2_ delivery to the peripheral muscles) (37, 38); (4) reduced cardiac output as a result of reduced pulmonary vascular volume and low left ventricle filling (leading to increased V/Q mismatch and physiologic dead space) (39); (5) altered afferents from pulmonary vessels and right heart due to increased pulmonary vascular pressures (40); and (6) increased sympathetic nervous system activation which increases chemosensitivity (41, 42). At this point, there is insufficient evidence to implicate the above-listed chemical factors in contributing to increased IND in mild COPD with the notable exception of decreased ventilatory efficiency which is likely to be important.

### Dynamic Respiratory Mechanics

Small airway dysfunction and obliteration is regarded as the hallmark feature of mild COPD and has important clinical consequences. Indeed, one study in symptomatic smokers who did not meet spirometric criteria for COPD showed significantly increased IND during exercise compared with healthy controls, in proportion to increased airway resistance (EMGdi) (43). In mild GOLD Stage 1 COPD, in mild GOLD Stage 1 COPD, EFL is variably present at rest, and often quickly becomes evident during the hyperpnea of exercise where it is associated with dynamic lung hyperinflation (DH) and its important negative sensory consequences (44). In some individuals with mild COPD, defined by spirometric criteria, localized emphysematous destruction of the lung's connective tissue matrix can alter lung elasticity (increase in lung compliance), resetting the balance of forces between inward lung recoil pressure and outward chest wall recoil at end-expiration. This results in an increased relaxation volume of the respiratory system (end-expiratory lung volume, EELV) compared with healthy controls. In patients with mild COPD exhibiting EFL at rest, dynamic EELV is influenced by the prevailing breathing pattern. If *F*_B_ increases abruptly (and expiratory time decreases and/or V_T_ increases) in patients with mild COPD and significant EFL, EELV temporarily and variably increases above its resting value (i.e., increased DH) during exercise. DH reflects the effect of the slow mechanical time-constant for lung emptying: expiratory time with each breath during exercise is simply of insufficient duration to allow EELV to decline to the predicted relaxation volume (14, 45–47).

The resting inspiratory capacity (IC) and inspiratory reserve volume (IRV) in mild COPD are generally preserved and V_T_ is therefore positioned on the more linear, mid-portion of the relaxed respiratory system sigmoidal pressure-volume relationship (48). However, during exercise when DH occurs, V_T_ becomes positioned closer to TLC at relatively low work rate which means that the inspiratory muscle fibers are shortened and functionally weakened and must contend with increased elastic mechanical loading. The difference between end-inspiratory lung volume (EILV) and TLC (i.e., IRV) largely dictates the relationship between IND and the mechanical/muscular response of the respiratory system and the degree of perceived dyspnea (49). Thus, the rate of dynamic decrease in IRV in mild COPD provides indirect information about the extent of neuromechanical dissociation of the respiratory system and has strong correlation with dyspnea intensity (49). Thus, when V_T_/IC ratio reaches ~0.7 during exercise or IRV reaches < 0.5–1.0 L), a widening disparity occurs between IND and the V_T_ response: IND continues to rise and V_T_ expansion becomes progressively constrained and eventually fixed, representing the onset of significant neuromechanical dissociation and escalation of dyspnea (50, 51).

Recent studies have clearly established that in mild COPD, reliance on traditional assessments of breathing reserve [estimated maximal ventilatory capacity (MVC) minus peak V_E_] can underestimate true ventilatory limitation indicated by premature attainment of critical respiratory mechanical constraints and accompanying severe dyspnea at relatively low work rates (10).

The question arises whether bronchodilator treatment, which partially reverses the above described abnormal mechanics, provides subjective benefits in patients with mild COPD. A study of symptomatic mild COPD patients showed that short-acting bronchodilators improved FEV_1_, reduced residual volume (RV) and resting airway resistance, DH, and work of breathing during exercise compared to placebo. However, there was no observed improvement in exercise endurance or exertional dyspnea, except at high V_E_ (52). A similar multi-center randomized double-blind study examining the effect of long-acting bronchodilator (tiotropium) in mild COPD patients showed a positive effect on resting and dynamic lung hyperinflation but with no improvement in exercise endurance or exertional dyspnea (53). The unimpressive effects of bronchodilators in mild COPD can be explained by the fact that resting IC is preserved in the majority of individuals. Thus, small improvements in dynamic respiratory mechanics at high ventilation levels near end-exercise are less likely to provide appreciable subjective benefit. Moreover, bronchodilators do not affect ventilatory inefficiency during exercise in mild COPD—a residual source of high IND and dyspnea (54).

## The Role of CPET in Uncovering the Cause of Activity-Related Dyspnoea

Accurate clinical interpretation of CPET requires comprehensive pre-assessment in each individual. A number of validated questionnaires are available to evaluate the extent of exertional dyspnea and its impact on quality of life and the patient's ability to undertake everyday physical activities (55, 56). Those with habitually reduced physical activity may have significant skeletal muscle deconditioning (a known contributor to higher ventilatory demand and increased dyspnea) (57). Documentation of comorbidities such as asthma, obesity, cardiocirculatory disorders, and musculoskeletal problems is also important. All patients with dyspnea disproportionate to spirometry should undertake additional tests such as single breath DLCO and plethysmographic lung volumes and resting arterial O_2_ saturation.

### CPET Interpretation: Panel Displays

**Figure 3:**
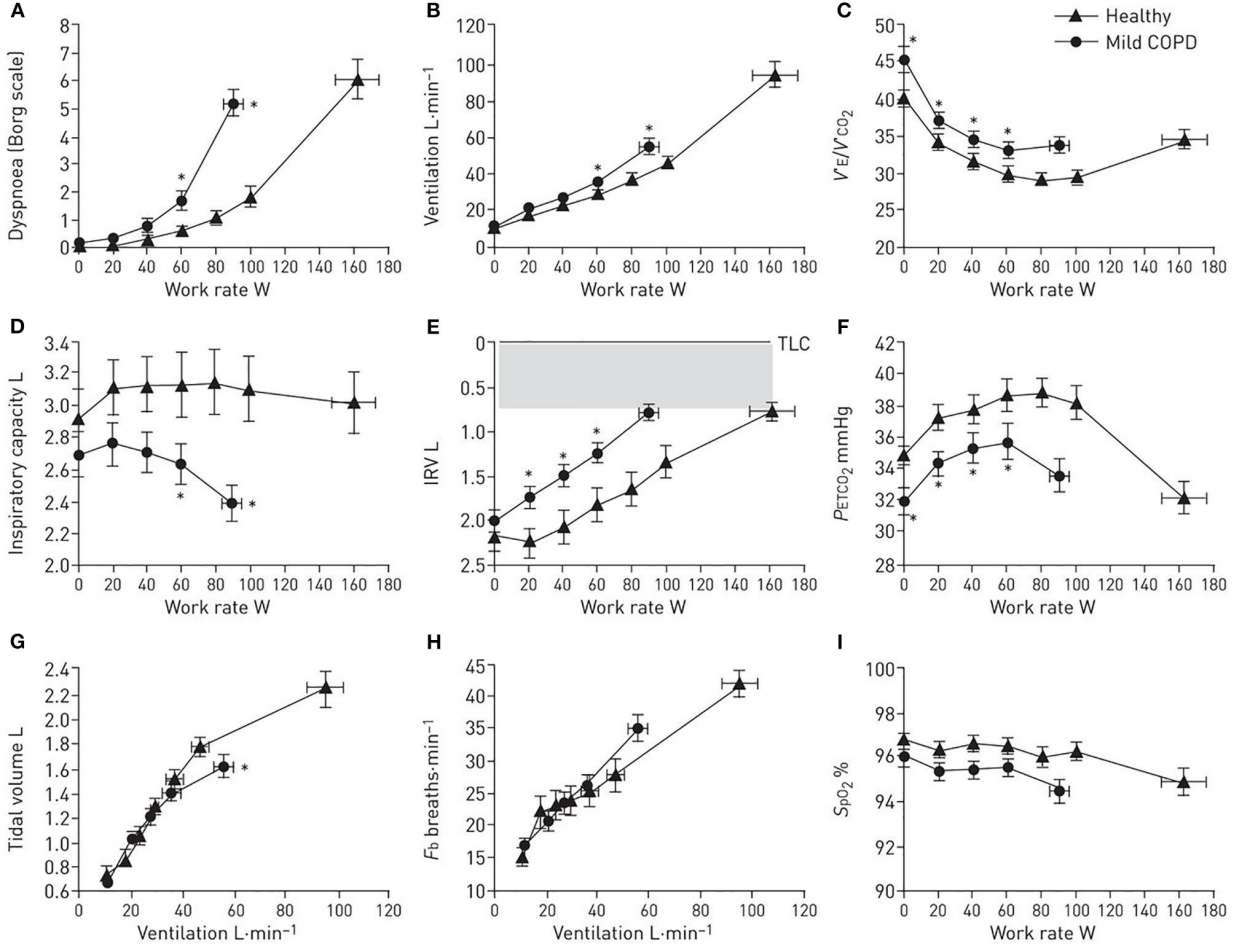
**(A–I)** Proposed panel displays during interpretation of an incremental cardiopulmonary exercise test. Data showing selected perceptual, ventilatory control, dynamic respiratory mechanics, and breathing pattern response to incremental cycle exercise in patients with mild chronic obstructive pulmonary disease (COPD) and age-matched healthy controls. Data are presented as Mean ± SEM. V_E_/VCO_2_: ventilatory equivalent for carbon dioxide; IRV: inspiratory reserve volume; *F*_b_: breathing frequency; P_ET_CO_2_: partial pressure of end-tidal carbon dioxide; SpO_2_: arterial oxygen saturation measured by pulse oximetry; TLC: total lung capacity. *p < 0.05 mild COPD versus healthy controls at rest, at standardized work rates or at peak exercise. Adapted with permission of the American Thoracic Society. Copyright © 2020 American Thoracic Society. All rights reserved. Cite: Author(s)/Year/Title/Journal title/Volume/Pages. The American Journal of Respiratory and Critical Care Medicine is an official journal of the American Thoracic Society. Readers are encouraged to read the entire article for the correct context at https://www.atsjournals.org/doi/full/10.1164/rccm.201211-1970OC The authors, editors, and The American Thoracic Society are not responsible for errors or omissions in adaptations.

To evaluate the magnitude of dyspnea intensity and exercise intolerance (at peak work rate or VO_2_) and identify potential contributing factors in mild COPD (Figure 3), the following responses are captured during incremental cycle CPET to tolerance: (1) *perceptual responses*: dyspnea (Borg) ratings as a function of work rate and/or V_E_; (2) *ventilatory control:* VO_2_/work rate, V_E_/work rate, (V_E_/VCO_2_)/work rate, O_2_ saturation (SpO_2_)/work rate, end-tidal CO_2_ (P_ET_CO_2_)/work rate; (3) *dynamic respiratory mechanics*: change in IC, IRV, V_T_, and breathing frequency (*F*_B_), all as a function of increasing work rate or V_E_; and (4) *cardiocirculatory responses:* heart rate relative to predicted peak heart rate and O_2_ pulse.

From this simple format, we can evaluate the extent of dyspnea and exercise intolerance in the individual. We can determine if ventilation slopes are increased relative to controls (indicating higher ventilatory drive) and enumerate its potential underlying cause(s) [e.g., increased ventilatory inefficiency (V_E_/VCO_2_), critical hypoxemia, or early ventilatory threshold]. We can also evaluate the extent of mechanical respiratory constraints (operating lung volumes, breathing pattern) (see also *Evaluation of dynamic respiratory mechanical abnormalities during conventional CPET*).

The dominant abnormalities in patients with mild COPD include: (1) increased IND secondary to high V_D_ as indirectly assessed by V_E_/VCO_2_ (higher nadir and steeper slope, see also *Measurement and interpretation of exercise ventilatory efficiency*) compared to age- and sex-matched healthy controls (Figure 3C); and (2) increased pulmonary gas trapping due to the combined effects EFL and increased ventilatory demand. The result is earlier critical mechanical constraints (reduced IC and IRV due to increased EELV, Figures 3D,E), and consequently higher exertional dyspnea ratings earlier in exercise (Figure 3A), compared to age-matched healthy individuals. In mild COPD patients free from significant ventilatory constraints at lower exercise intensities, the measured V_E_/VCO_2_ (higher V_E_/VCO_2_ nadir and steeper V_E_/VCO_2_ slope compared to health) is a reliable surrogate for increased physiological dead space (58).

#### Influence of Co-morbidities in COPD CPET Interpretation

In smokers with persistent dyspnea but unremarkable spirometry there is justifiably a high index of suspicion for cardiovascular dysfunction which usually prompts a cascade of investigations to rule out active ischemic heart disease. The cardiocirculatory responses during CPET indicating concomitant left ventricular dysfunction include high V_E_/VCO_2_ nadir, uniformly low P_ET_CO_2_, relative tachycardia, reduced O_2_ pulse (VO_2_/HR), reduced VO_2_/work rate slope, and early ventilatory threshold and complaints of dominant leg discomfort indicating impaired oxygen delivery to the peripheral muscles. It must be remembered that profound skeletal muscle deconditioning as a result of longstanding avoidance of physical activity can present with similar physiological responses to exercise as those encountered in patients with reduced cardiac output. Relevant clinical history and the incorporation of 12-lead ECG into CPET to detect undiagnosed ischemic heart disease in these patients can be informative. The findings of very high V_E_/VCO_2_ nadir (>35), arterial O_2_ desaturation together with physiological features of LV dysfunction (listed above) raises the possibility of pulmonary arterial hypertension which should prompt further relevant investigations.

Obesity also influences exercise responses in COPD in a manner that is readily discernable (50, 59). There is an upward parallel shift in the VO_2_/WR slope, explained by the increased metabolic requirement of lifting heavy limbs during cycling. Obese COPD patients have an increased resting IC (and thus lower EELV) which means that the patient can exercise to a higher ventilation before the V_T_ plateau or minimal IRV is reached (60). In severe obesity, IC becomes eroded due to mass loading effect and reduced respiratory system compliance, pulmonary gas exchange becomes compromised, PCO_2_ may not decline or actually increase during exercise, and the load/capacity imbalance of the respiratory muscles reaches a critical level (59). These patients often attain physiological limits of the respiratory system and distressing dyspnea at relatively low exercise levels compared with those with normal weight COPD (59, 60).

### Clinical and Therapeutic Implications

CPET uniquely exposes the nature and extent of physiological impairment that can exist in individual smokers with mild COPD who present to the clinician with troublesome symptoms of dyspnea and exercise intolerance. The new CPET-derived information alerts the clinician of the importance of careful follow-up to monitor disease progression and may persuade the patient to live a healthier lifestyle and avoid further lung injury from tobacco smoking. While CPET can provide a deeper understanding of the mechanisms of dyspnea in the individual, patients, management options are currently limited beyond smoking cessation, weight reduction (when appropriate) and encouragement of regular physical activity. In the setting of mechanical abnormalities such as dynamic lung hyperinflation during exercise, a trial of a short-or long-acting bronchodilator seems warranted even as we await definitive evidence from future clinical trials of bronchodilator efficacy in mild COPD populations. At present, no treatment options are available for a minority of symptomatic patients with mild COPD who manifest features of “a microvascular phenotype” (low DLCO; high V_E_/VCO_2_ nadir, with or without minor centrilobular emphysema on CT) (61). Future studies that elucidate the pathogenesis of vascular injury in smokers with mild COPD will hopefully lead to the development of new targeted therapies. Finally, CPET may help identify clinically important co-morbid conditions in patient with Mild COPD that require specific therapeutic interventions.

## Conclusions

“Mild COPD” is in many respects a misnomer as it mainly signifies presence of mild airflow limitation, measured by simple spirometry. It is now well-established that such individuals often have persistent troublesome symptoms, and consistently report reduced physical activity and poor health status. We now know that spirometry, while useful for defining severity of airflow limitation, can obscure extensive and heterogeneous physiological impairment of the lungs that additional tests and modern imaging techniques can reliably uncover. CPET, which examines the respiratory system under stress, is uniquely positioned to expose, in an integrated manner, the nature and extent of physiological impairment, and its negative sensory consequences. The most consistent abnormalities in mild COPD during exercise are high inspiratory neural drive, reduced ventilatory efficiency, and early dynamic mechanical constraints to increasing ventilation as metabolic demand increases. Patients with unremarkable spirometry (and normal arterial O_2_ saturation during walking) who have DLCO < LLN and high V_E_/VCO_2_ nadir during CPET, are more likely to experience dyspnea and reduced exercise tolerance than those who do not exhibit these features. While CPET results do not currently influence therapeutic choices on an individual basis, the information obtained provides the clinician with valuable insights into the underlying causes of exertional dyspnea in mild COPD. Moreover, CPET is potentially important in helping to define discreet physiological phenotypes of COPD (e.g., dominant microvascular dysfunction, dominant small airway dysfunction, or mixed patterns) linked to clinical outcomes. This type of deep physiological phenotyping could allow more refined structure-function analysis to ascertain the clinical relevance of morphometric abnormalities revealed by novel imaging. Ultimately, such phenotypic characterization of individuals which incorporates CPET, has the potential to guide more precise interrogation of the pathobiology of COPD to hasten the discovery of new therapeutic targets for this complex disease.

## Author Contributions

All authors played a role in the content and writing of all sections of the review. In addition: DO'D provided the original idea for the review. All authors contributed to the article and approved the submitted version.

## Conflict of Interest

Outside of this review, DO'D has received research funding via Queen's University from Canadian Institutes of Health Research, Canadian Respiratory Research Network, AstraZeneca, and Boehringer Ingelheim and has served on speaker bureaus, consultation panels, and advisory boards for AstraZeneca and Boehringer Ingelheim. MJ was supported by anOntarioGraduate Scholarship. The remaining authors declare that the research was conducted in the absence of any commercial or financial relationships that could be construed as a potential conflict of interest.
